# Phylodynamics and Dispersal of HRSV Entails Its Permanence in the General Population in between Yearly Outbreaks in Children

**DOI:** 10.1371/journal.pone.0041953

**Published:** 2012-10-15

**Authors:** Hagit Katzov-Eckert, Viviane F. Botosso, Eurico Arruda Neto, Paolo Marinho de Andrade Zanotto, and the VGND consortium

**Affiliations:** 1 Laboratory of Molecular Evolution and Bioinformatics, Department of Microbiology, Biomedical Sciences Institute-ICB-II, University of São Paulo, São Paulo, Brazil; 2 Butantan Institute, Virology Branch, Butantã, São Paulo, Brazil; 3 Department of Cell Biology, School of Medicine of Ribeirão Preto, University of São Paulo, Ribeirão Preto, São Paulo, Brazil, and the VGDN Consortium; Columbia University, United States of America

## Abstract

**Background:**

Human respiratory syncytial virus (HRSV) is one of the major etiologic agents of respiratory tract infections among children worldwide.

**Methodology/Principal Findings:**

Here through a comprehensive analysis of the two major HRSV groups A and B (n = 1983) which comprise of several genotypes, we present a complex pattern of population dynamics of HRSV over a time period of 50 years (1956–2006). Circulation pattern of HRSV revealed a series of expansions and fluctuations of co-circulating lineages with a predominance of HRSVA. Positively selected amino acid substitutions of the G glycoprotein occurred upon population growth of GB3 with a 60-nucleotide insertion (GB3 Insert), while other genotypes acquired substitutions upon both population growth and decrease, thus possibly reflecting a role for immune selected epitopes in linkage to the traced substitution sites that may have important relevance for vaccine design. Analysis evidenced the co-circulation and predominance of distinct HRSV genotypes in Brazil and suggested a year-round presence of the virus. In Brazil, GA2 and GA5 were the main culprits of HRSV outbreaks until recently, when the GB3 Insert became highly prevalent. Using Bayesian methods, we determined the dispersal patterns of genotypes through several inferred migratory routes.

**Conclusions/Significance:**

Genotypes spread across continents and between neighboring areas. Crucially, genotypes also remained at any given region for extended periods, independent of seasonal outbreaks possibly maintained by re-infecting the general population.

## Introduction

Human respiratory syncytial virus (HRSV) causes serious respiratory tract infections in infants, elderly and immunocompromised adults [1], [2], [3], [4]. HRSV epidemics are associated with climate patterns and occur annually in late autumn and winter in temperate climates, and within the rainy season in tropical countries [5], [6]. It is estimated that 64 million HRSV infections occur annually, resulting in 160,000 deaths (Initiative for Vaccine Research: respiratory syncytial virus, World Health Organization http://www.who.int/vaccine_research/diseases/ari/en/index3.html, update September 2009). Children are susceptible to repeated HRSV infections and to developing severe disease [7], [8]. In 2005, an estimated 33.8 million HRSV-associated acute lower-respiratory tract infections (ALRTI) occurred in children under five years of age; 3.4 million cases required hospital admission and 66,000–199,000 children died [9]. Reports on HRSV in developing countries have shown that HRSV-related mortality is higher than in industrialized countries [5], [10].

In Brazil, 30–50% of outpatient consultations and more than 50% of hospitalizations are attributed to ALRTI [11]. Strikingly in Brazil, 10–15% of deaths of children under five years old were attributed to ALRTI, 80% of which were due to pneumonia, and 22–38% have been associated with HRSV infections [12], [13], [14], [15], [16], [17].

HRSV is an enveloped non-segmented, non-recombinant in nature, negative RNA virus, classified within the *Paramyxoviridae* family [18], [19], [20]. Two major groups of HRSV have been described based on antigenic and genetic studies, HRSVA and HRSVB [21], [22], [23], [24]. Both groups co-circulate in each epidemic period. Genomic characterization has further divided HRSVA and HRSVB into genotypes: GA1–GA7, and SAA1 [25], [26], GB1–GB4, SAB1–SAB3 and GB3 with a 60-nucleotide insertion (GB3 Insert [25], [26], [27]. These genotypes can co-circulate in the same community, usually with a predominance of one or two genotypes, which can shift over the years [28], [29], [30], [31], [32], [33], [34], [35], [36], [37], [38], [39]. The G glycoprotein is one of the main antibody target responsible for neutralizing immune responses to HRSV [22] and displays extensive heterogeneity between and within genotypes [40], [41], [42], [43].

The G protein is a type II integral protein of 289 to 319 amino acids, depending on the viral strain [44]. The attachment G protein can be divided into an intracellular domain, a transmembrane domain, and an ectodomain. The ectodomain is comprised of two hypervariable mucin-like regions (HVRs) which are extensively glycolylated with both N- and O-linked sugars and contain a high proportion of proline [45]. The HVRs are separated by a highly conserved non-glycolylated region comprising residues 151–190 which contains four cysteines held together by disulfide bonds in a cysteine noose that assumed to represent a receptor-binding site [46]. The HVRs are under positive selection [47], [48] and contain multiple epitopes that are recognized by both murine monoclonal antibodies and human convalescent sera [49].

The variability between HRSV genotypes is one of the features of HRSV infections that contribute to the ability of the virus to infect people repeatedly and cause yearly outbreaks [50], [51]. HRSV variants are under constant pressure from the human immune response [52], [53], [54]. Possibly, human immunity influences HRSV evolution and selects which genotypes will predominate in consecutive seasonal outbreaks [47]. To date there is no effective vaccine available against the virus [55].

Based on the coalescent theory of Kingman (1982), intra-species gene genealogies have been extensively used to infer various demographic parameters for a diverse set of organisms [56], allowing statistical inferences to be made on the time and mode of evolution of a diverse set of organisms ranging from endogenous [57] and exogenous viruses [58], [59], [60] up to complex metazoa [61]. Notably, results obtained from the genealogies and epidemiology of Dengue viruses showed almost precise agreements between the dynamic patterns over time estimated, which suggest that phylodynamics can recover equivalent information to that obtained by the number of notified cases through time in an outbreak [62]. To contribute to our understanding of HRSV, particularly its dynamics and spatial patterning we performed an evolutionary analysis on available HRSV genome sequences sampled globally.

## Results

**Figure 1:**
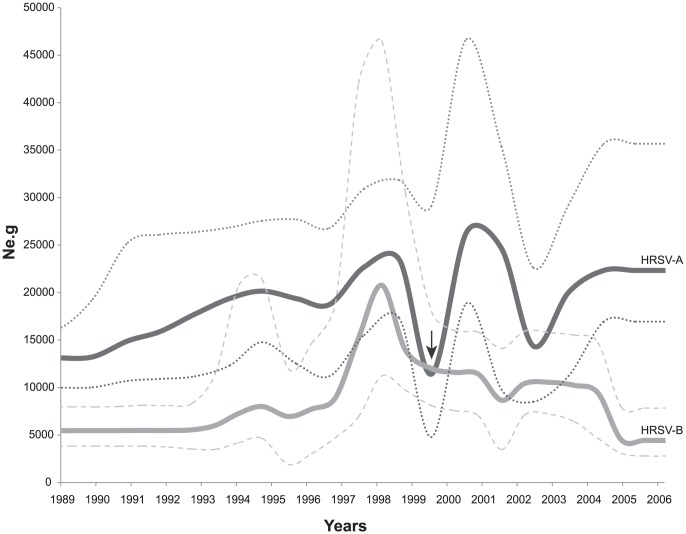
Global demographic history of HRSV genotypes. Bayesian skyline plots of complete HRSV sequences of HRSVA (n = 1203) and HRSVB (n = 780). The *y*-axis represents a measure of relative genetic diversity presented as *Ne.g* reflecting the change in effective population (a surrogate for number of infections) over time for the complete set of HRSV sequences for HRSVA (n = 1204) and HRSVB (n = 778). The dotted lines define the likelihood bounds corresponding to a 95% confidence interval (CI). (…) - Upper and lower limits for HRSVA; (—) - Upper and lower limits for HRSVB. The arrow represents a shift in dynamics between HRSVA and HRSVB.

**Figure 2:**
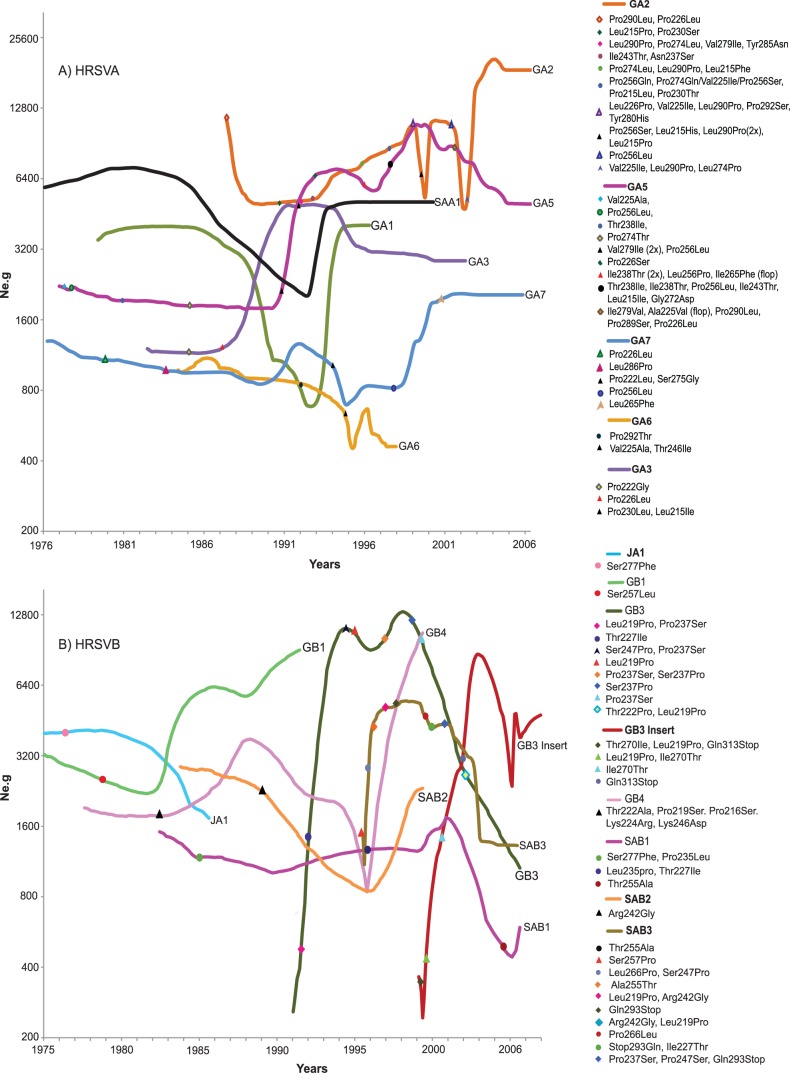
Population dynamics and genetic diversity of HRSV. A) Bayesian skyline plots of HRSVA genotypes B) Bayesian skyline plots of HRSVB genotypes. Positively selected amino acid substitution sites are represented as previously described by Botosso et al. [48]. The *y*-axis represents a measure of relative genetic diversity presented as *Ne.g* reflecting the change in effective number of infections over time.

Sequences of the variable region 2 of the G glycoprotein (G2) gene of HRSVA (n = 1203 sampled globally between 1956 and 2005) and HRSVB (n = 780 sampled globally between 1960 and 2006) were used to classify sequences into distinct genotypes consistent with previously assigned nomenclatures [48] (viral genealogies available from the authors upon request). Bayesian skyline plot (BSL) analyses were performed to reconstruct the past population dynamics of HRSV (Figures 1 & 2), while also portraying its evolutionary dynamics by examining the process of amino acid replacements and changes in effective population size (*Ne.g*) of genotypes over time (Figure 2).

### Phylodynamics of HRSV

The Bayesian skyline of global HRSV gene sequences showed a predominance of HRSVA over HRSVB during the entire period of the analysis, with the exception of 1999, when the two BSL plots crossed one another (Figure 1). Initially, a steady demographic expansion with slight fluctuations of both major HRSV groups (HRSVA and HRSVB) was observed between the years 1990 and 1998, with a dramatic fluctuation between mid-1998 to 2000. Sampling of HRSV steadily increased from 1980, and the majority of samples were collected between 1996 and 2004 (for HRSVA) and between 1996 and 2005 (for HRSVB) (mean = 1999; Figure S1). In the years between 2000 and 2002, HRSVA had its highest value of effective population (*Ne.g*), coinciding with HRSVB's slow decrease. Both HRSVA and HRSVB circulation fluctuated between 1996 and 2005. Importantly, the circulation of HRSVA was positively correlated with that of HRSVB before 1999, and became negatively correlated thereafter (see vertical arrow in Figure 1 and Figure S2). In addition, in very recent years the circulation of HRSV groups A and B was positively correlated, yet with a very low signal, corresponding to the flattening of the skyline observed in the dynamics of HRSVA and HRSVB after 2005.

### Phylodynamics of HRSVA genotypes

**Figure 3:**
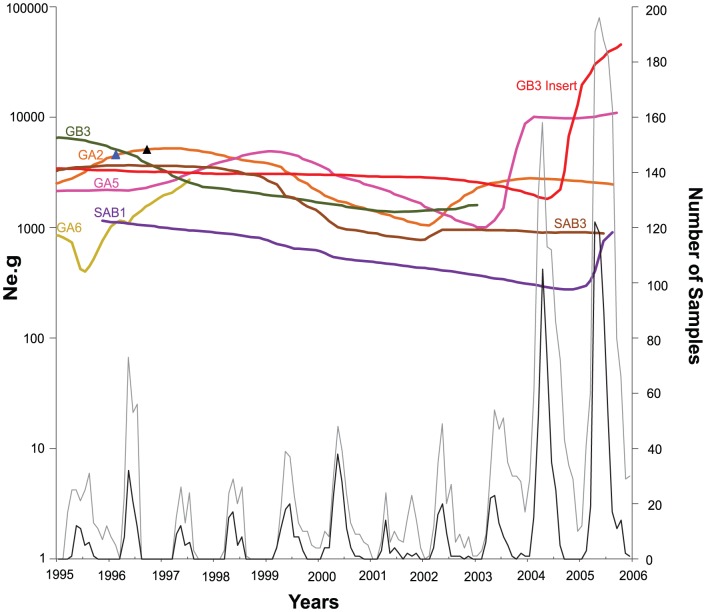
Epidemiology and population dynamics of HRSV in São Paulo. Bayesian skyline plots of HRSV genotypes prevalent in São Paulo (top) and seasonal distribution of HRSV cases in São Paulo during the 1995–2005 seasons are shown in the *x*-axis. GA1, GA3 GA7 and GB4 were excluded from the BSL analysis because of small sample size (n<10). The *y*-axis (on the left) represents a measure of relative genetic diversity presented as *Ne.g* reflecting the change in effective number of infections over time; where *g* is the average generation time. The *y*-axis (on the right) represents the number of samples in the study. (-) - Number of total samples collected during the period. (-) - Number of all HRSV positive cases identified with monoclonal antibodies and molecular characterization.

Genotypes GA2 and GA5 appeared to have predominated HRSVA outbreaks since the beginning of the 1990s (Figure 2). In 1991, an increase in the effective population (*i.e. Ne.g*) of GA5 was observed modulating its circulation pattern with GA2. By 2002, GA2 had the higher population growth rate (Figure 2a). These results agree with reports showing GA2 and GA5 to be the most prevalent HRSVA genotypes of contemporary times [33], [35], [63], . Our previous analysis of positive substitution sites for HRSVA indicated that population growth coincided with an increase in the number of replacements of amino acids in the G2 protein on sites described to be under positive selection pressure for both genotypes (GA2, GA5; Figure 3a [48]). Notably, immunologically important amino acid substitutions (Pro290Leu, Pro274Leu, Val225Ile, Leu226Pro and Pro256Leu, or Ser or Gln [42], [67], [68] were observed in GA2 upon its increase, as well as several substitutions upon its decrease (Pro256Ser, Ile238Thr and Leu256Pro) (Figure 2a). A substitution of Leu to Pro on site 274 in 1996, and a reversion of this site from Pro to Leu in 2002 on a monophyletic lineage within GA2 (characteristic of a ‘flip-flop’ pattern [48]) corresponding to an increase in GA2 was also observed. There were many ‘flip-flop’ substitutions in monophyletic lineages of GA5 especially upon its increase between 1995 and 1998 (Ile238Thr and Leu256Pro). A correlation between amino acid replacements and dynamic changes for the minor HRSVA genotypes was not evident. Furthermore, a flat BSL for HRSVA genotypes between 1976 and 1985 was observed, possibly indicative of stable dynamics and/or loss of cladogenetic signals due to coalescence and loss of older lineages that were not sampled. Lineages, GA1, GA3, GA6 and SAA1 appeared to have been either vanished or stopped being sampled at different times. Interestingly, several of the minor genotypes appeared to maintain stable endemicity through time periods spanning across seasonal outbreaks (GA3, SAA1, and GA7).

### Phylodynamics of HRSVB genotypes

HRSVB predominant genotypes replaced each other over time. JA1 was followed by GB1 in the early 90s. From the mid-90s onwards, the three main HRSVB genotypes circulating globally were GB3, GB3 Insert, and SAB3 (Figure 2b). After 1991, GB3 expanded rapidly, reaching a peak in 1998 before decreasing between 2001 and 2005. Similarly, SAB3 related to GB3 [26], [48] was first detected in 1995, grew rapidly until it reached a peak in 1998 and then began to fluctuate. Subsequently in 2002, SAB3 decreased sharply. Interestingly, fluctuations in both GB3 and SAB3 were followed by several G2 glycoprotein amino acid substitutions (Figure 2b). These substitutions have been previously proposed to be under positive selection in both genotypes [48]. Demographic data inferred from the BSL plots showed that for three years between 1996 and 1999 SAB2, SAB3, and GB4 all appeared to have experienced rapid growth. A noticeable decrease in *Ne.g* for SAB1, as well as for GB3 and SAB3 coincided with the rapid expansion of GB3 Insert followed by amino acid substitutions in sites 270(Thr→Ile), 219(Leu→Pro) and 313(Gln→stop), from 2001 to 2003 in GB3 Insert (Figure 2b). In addition, during the fast growth of the GB3 Insert (see its skyline plot in Figure 2b) the Thr270Ile substitution in 1999 was followed by its reversal (Ile270Thr) in 2001, as previously reported [48]. It is still unknown whether these sites are immunologically important since HRSVB epitopes are not as well characterized as HRSVA epitopes. Furthermore, an expansion in the effective population of the GB3 Insert was observed followed by drastic fluctuations and bottlenecks near the present [69], [70]. These data along with results obtained from previous phylogenetic analyses [48] suggest that the bottleneck effect on the population size could be related to the predominance of the GB3 insert over the other HRSVB genotypes [71], [72]. In summary, drastic fluctuations in the BSL were noticed for the main genotypes of both HRSVA and HRSVB groups. We argue that this behavior, possibly made more evident due to denser sampling near the present, may be suggestive of a characteristic logistic growth bound by asymptotic behavior, in which population grows to a certain size and then either remains constant or produces oscillations [73].

### Phylodynamics of HRSV in Brazil

The BSL plots for Brazilian HRSV genotypes, include those sampled during eleven consecutive years (1995–2006) in São Paulo State are shown in Figure 3 (additional detailed epidemiological data for patients in São Paulo are described in Botosso et al. unpublished data for the 1995 to 2006 seasons; Vieira et al. [17] for the 1995 and 1996 seasons; and Oliveira et al. [74] for the 2003 to 2006 seasons). Data indicated an increase in *Ne.g* of GA2 in 1995. We also observed a similar yet delayed increase of GA5 (Figure 3). From then on, both genotypes decreased between 1997 and 2002. Subsequently, between 2002 and 2004, GA2 increased again before stabilizing. Importantly, Botosso et al. [48] showed that several GA2 lineages incorporated amino acid substitutions: Leu215Pro, Arg244Lys, His266Tyr, Asp297Lys, Stop298Trp that were fixed in all Brazilian GA2 strains circulating between 2003 to 2005 (Figure 3 top). GA5 increased rapidly from 2003 to 2004 before stabilizing in 2005. In this final phase, both genotypes had alternating predominance. Lastly, genotype GA6 fluctuated in its dynamics until 1997, the last year that GA6 was sampled from our HRSV infected patients.

Similar to HRSVA genotypes, GB3, SAB1 and SAB3 circulated together in the mid-1990s (Figure 3 top). At first GB3 was the predominant HRSVB genotype circulating in the region, although both GB3 and SAB3 had a gradual decrease in *Ne.g* until recently. The GB3 Insert had its time to the most recent common ancestor (TMRCA) in 1998 and showed a sharp growth around 2004, thenceforth becoming the predominant HRSV genotype to circulate in the region. A moderate increase in the effective population of SAB1 in 2005 was also observed. Further evidence for circulation patterns of genotypes was sought with cross-correlation analysis of one-year circulation cycles in São Paulo State. Analysis revealed significant positive cross correlation between GA2 and SAB1; GA2 and SAB3; GB3 and SAB1; GB3 and SAB3; SAB3 and SAB1. Additionally, though not significant, the circulation of GA2 and GA5, and the circulation of GB3 Insert and SAB1 were positively correlated (data not shown).

### Phylogeography of HRSV

**Figure 4:**
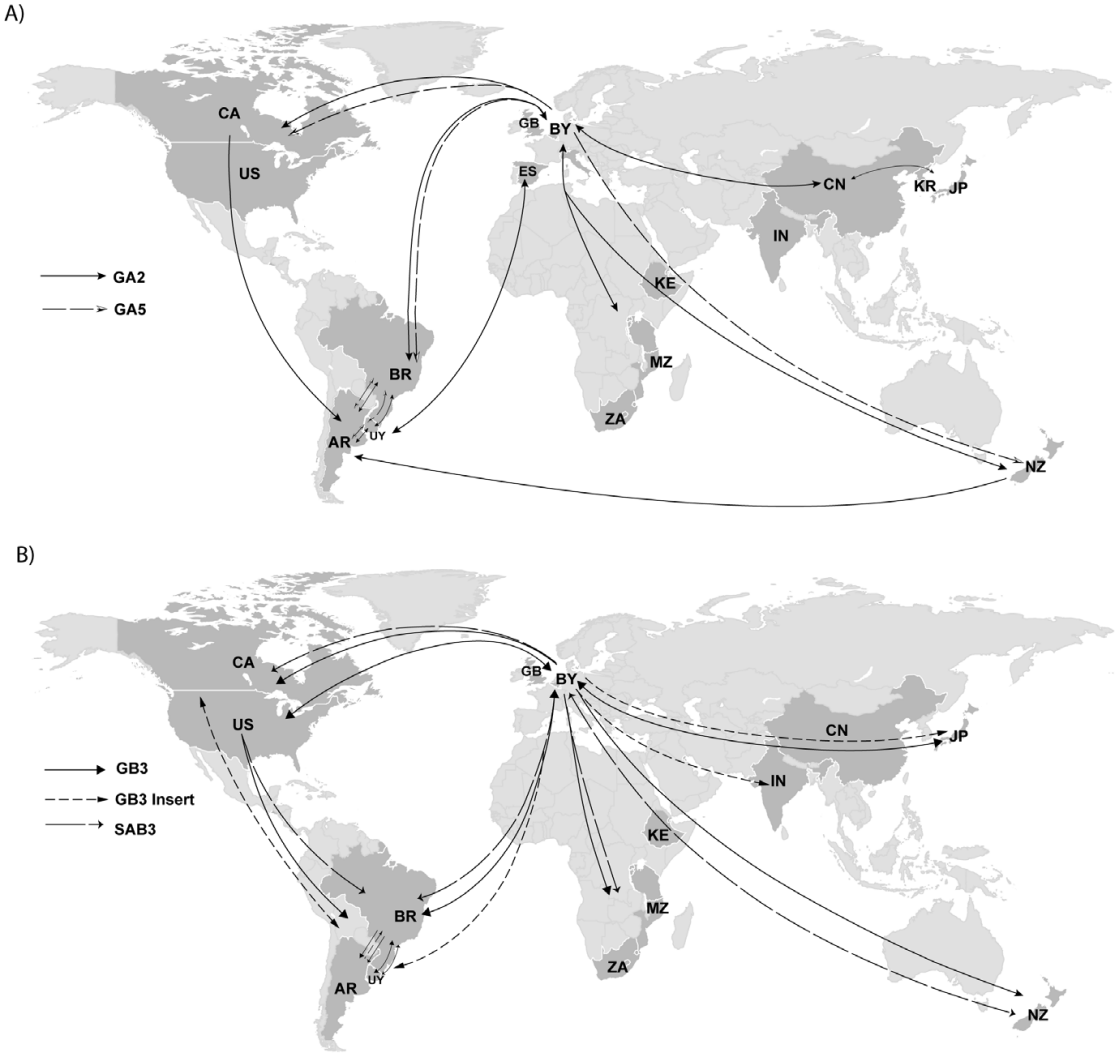
Plausible migration routes of HRSV. A) Major HRSVA genotypes B) Main HRSVB genotypes c) Minor and sporadic HRSV genotypes. Migration routes and directionality were discerned from MCC phylogenetic trees and Bayes factor rates.

HRSV genotypes displayed a complex dispersal pattern reaching distant places in a relatively short period of time (Figure 4). The maps in Figure 4 represent a summary of exchanges among continents with Bayes factor (BF) rates above 3, and additional exchanges that were evident from the maximum clade credibility (MCC) trees for 5 genotypes of HRSVA and 5 genotypes of HRSVB (Figure S3). In detail, GA2 appeared first in Oceania and spread to Europe (BF = 2634.2) and South America. High Bayes factor rates (BF = 1975.1) indicated that there were exchanges of GA2 between neighboring countries in South America. Europe possibly acted as a hub for the spread of GA2 to different regions. In fact, the genotype was in Europe for a long period of time (from the 1980's to 2005), indicating that GA2 circulated in the region throughout several yearly seasonal cycles. Similarly to the dispersal of GA2, Europe appeared to act as a reservoir for GA5 throughout the period under study. The source of global spread of GA5 could not be determined since the MRCA (Most Recent Common Ancestor) was possibly biased from the oldest samples from North America. Bayes factor rates showed evidence of exchanges of GA5 between Europe and South America (BF = 2670.4), between Europe and Oceania (BF = 1779.55) and between neighboring countries in South America (BF>10690.16).

Movement of GB3 indicated that Europe was a source of multiple independent entries of the genotype into South America (BF = 27.8), Oceania and Africa in the 1990's. Analyses on GB3 Insert showed a bias in the MRCA towards the oldest samples from Asia though data indicated that the Insert spread from South America and Europe to different regions. Significant exchanges were noticeable for GB3 Insert between North and South America (BF = 41.3) and within neighboring countries in South America (BF>8175.71). SAB3 spread worldwide from Oceania and Europe in the mid-1990s. Both Europe and South America may have acted as major hubs for the spread of SAB3 with multiple exchanges between the continents throughout the period under study (BF = 18.86). The highest BF rate for SAB3 was identified for exchanges between North and South America (BF = 165.2). Even-though we had reduced number of sequences of the minor (GA6, SAA1, SAB1, SAB2) and sporadic (GA7, GB4) genotypes, which possibly caused the unveiled spread to have lower BF for their rates, the adjacency patterns on the MCC trees revealed that these genotypes were also introduced to most continents (Figure 4).

## Discussion

The high rate of evolutionary change in HRSV may confer selective advantage and facilitate re-infections of the virus. Genotypes accumulating several mutations have been predicted to be the precursors of new lineages due to antigenic drift for the influenza virus [58], [75]. Nevertheless, there is still a need to better understand how the complex interactions among HRSV lineages in its human host may affect its pattern of genotype replacement in space and time. While trying to address some of these issues, this study provides a large-scale phylogeographic analysis establishing the divergence process and demographic history of HRSV genotypes. Our analyses further showcased both the differences in the relative genetic diversity and the patterns of co-circulation between HRSV genotypes and revisited the associated genotype replacement process [48].

### The Phylodynamics of HRSV

From the demographic history of HRSV (Figure 1) a ‘*millennium shift*’ was observed whereby at the end of the 1990s there was an apparent significant shift in cross-correlation between the overall dynamics of HRSVA and HRSVB. Interestingly, at the same time GA1, GA6 and SAB2 apparently disappeared, GB3 Insert globally increased after 2001. It is worthwhile to consider that a lack of sampling of any given HRSV genotype does not mean that the lineage itself may have necessarily died out, since it may be experiencing cryptic circulation in the regions that we examined, or it may be still circulating in other localities that we did not obtain samples from. Nonetheless, the dynamics profile of the GB3 Insert indicated a clear pattern of population growth concurrent with amino acid replacements at positively selected codons of the immunogenic G glycoprotein (as well as a significant change by the addition of 20 amino acids to the variable region of the protein). This pattern was not always the case for other HRSVB genotypes (Figure 2). Particularly, we observed considerable amino acid substitutions in GB3 and SAB3 under positive selection, which appears to be at odds with the notion of immune escape, since these two genotypes were subsequently replaced by the GB3 Insert (Figure 2b). These observations deserve some consideration. There appears to be a consensus on the notion that replacements coinciding with viral population expansions are selectively advantageous and possibly associated with escape mutations [75]. On the other hand, those positively selected substitutions observed upon population reduction are no less interesting. We argue that these positively selected substitutions may reflect the complex role of immune reactive sites outside the sites we looked upon at the variable G2 domain in the G glycoprotein, and that they may also be under linkage with the substitution sites that we traced, such as the major neutralizing antibody epitope that sits between amino acid residues 150 and 170 at the cysteine noose domain [76]. We further argue that this ‘linked selection’ effect would depend on a large viral population [77] and the scarcity of recombination events, which is precisely the case for nonsegmented negative-strand RNA viruses (*Mononegavirales*) in general, and for HRSV in particular [20]. Under this scenario, the reductions in *Ne.g* following amino acid replacements observed could reflect the outcome of viral strain competition under human immune surveillance. Apparently the ‘flip-flop’ reversals that we observed may be only an example of a more general process. Recent studies have shown that a cyclic arms race between viruses and mammal hosts takes place under conditions where viruses are relatively constrained. A ‘rock-paper-scissors game’ model remarkably similar to the ‘flip-flop’ pattern we observe in HRSV has been proposed, which explains the co-evolution of several genes, such as, Nef, TRIM5 and Vif interacting with retroviral capsid of the Simian immunodeficiency virus (SIV) and APOBECC3G interacting with HIV and SIV [78].

In Brazil, the typical seasonal epidemic outbreak of HRSV with the expected sharp yearly peaks (Figure 3, Botosso et al. unpublished data, [17], [74]), together with the estimated relative population growth of genotypes (Figure 3) reflect the maintenance of lineages in the population, as demonstrated in several hospital-based longitudinal studies [5]. A similar pattern has also been observed for the influenza virus in tropical regions [79], [80], [81]. Asymptomatic adults are most likely the source of the viral reservoir that determines seasonal outbreaks in immunologically naïve populations of children [82]. This inconspicuous group of carriers may not only be a main HRSV reservoir, but also the determining factor governing its long-term dynamics that does not seem to be affected by the yearly cyclic outbreaks we observed among children (Figure 3). Accordingly, we clearly observe a trend in the gradual shifting of predominant genotypes in the demographic history at a slower pace, sometimes taking several years encompassing successive HRSV seasons (Figure 2). This suggests that perhaps herd immunity to HRSV may have a significant role in the shifting of the predominant genotypes throughout successive epidemics [82], [83].

### HRSV spread in space and time

Our G2 gene genealogies suggested that the HRSV genotypes we sampled were introduced from Europe into the Americas. The pattern indicated a movement of genotypes from the Northern to the Southern hemisphere, which agrees with the fact that the majority of the human population resides in the Northern hemisphere. Substantially more people engage in international and transcontinental travel, which may explain why HRSV appeared to have entered Asia via Europe and Oceania [83], [84]. We also observed back and forth movement of genotypes between Europe and Oceania, which has also been observed for other respiratory viruses, such as influenza [85], possibly as a result of stronger socio-economic ties within these regions [83], [84], [86]. There were numerous exchanges of genotypes within neighboring countries in South America. Furthermore, HRSV dispersal inferred across the gene genealogies showed that Oceania appeared to be a geographic source for multiple genotypes (GA6, GA7 SAA1, SAB1, SAB2 and SAB3). Interestingly, of those genotypes GA6, SAB2 and SAA1 have not been sampled recently (Figure 2). Given the dense sampling we had for Europe and South America, we could see these localities acting as major hubs for the worldwide spread of HRSV during extended periods. Another potential bias may have been introduced by using different sampling timeframes for the different continents. These sampling biases that were instrumental in establishing the residence time of genotypes in a given locality, could have also introduced biases on the *directionality* of the recovered migratory events. Although there is no clear route of HRSV migration, the dispersal of the virus does fit well with the pattern of human movement along major travelling routes (Figure 4, see also Figure S4 Google Earth ‘movies’ showing worldwide HRSV spread in time and space). Within genotypes, clusters of sequences sampled from the same location were observed, but these clusters were interspersed with those from other locations. This pattern suggests a geographic genetic flow, whereby not only distinct genotypes endemically persist in diverse parts of the world but also the movement of viral lineages among locations [41], [87], [88]. Although this phylogenetic pattern may be affected by inconsistent sampling through the years, it may also indicate HRSV's temporal strain replacement occurring globally, which agrees with the observed changes in their cyclical clinical prevalence. On the other hand, it is important to stress that the span of GA2 and GA5 in particular and of several other genotypes in space and time demonstrate findings that HRSV may *remain* in a regional hub while being continuously broadcasted elsewhere for a long period of time, independently of the pattern of seasonal outbreaks.

Crucially, if the main component in HRSV transmission were the observed seasonal outbreaks occurring among children we could expect to observe random fluctuations between seasons in the overall HRSV genotypes causing fast succession of genotypes (*i.e.*, clade replacement), possibly on a yearly basis [83], [89]. Essentially, since our results indicate that HRSV subtypes remain monophyletic at the same place for long periods of time, a plausible explanation for these findings may be that the virus remains in the general population since the virus has no other host. We also show the introduction of new epidemic strains around the globe which makes persistence, introduction and re-introduction important mechanisms for HRSV maintenance. Accordingly, simulation studies have shown that introduction of new infections in a community may be necessary for the persistence of HRSV in a population [90]. The transmission dynamics of HRSV is complex and both asymptomatic carriers and influx of infections allow for the maintenance and for the occurrence of epidemics in a community. Eventhough we used all available data, a limiting factor of our study is that sequences were only from a certain number of locations with sparse and time-restricted sampling. Nonetheless, the pattern of the underlying demographic history unfolded by our coalescent-based analysis is one of slow dynamics characterized by a series of slow sequential expansions and fluctuations, evidencing the replacement of predominance among lineages that have otherwise a long-term permanence (*i.e.*, circulation) in the population.

## Materials and Methods

### Nucleotide sequences

Partial HRSV G gene sequences (from Brazil (n = 568) and from elsewhere (n = 1415)) with known collection dates between 1956 and 2006 were used in this study. A comprehensive list of groups of sequences analyzed along with Genbank accession numbers, geographic origin and sample collection date are provided in Table S1 and Table S2.

### Respiratory samples

Clinical samples from Brazil (n = 3695) were collected from infants and young children from 1 week to 5 years of age, hospitalized with acute lower respiratory tract infection (ALRTI) in the state of São Paulo, Brazil over eleven consecutive HRSV seasons from 1995 to 2006 (Botosso et al. unpublished data). Description of sample typing methods and ALRTI patients has been reported previously [14], [48], [74]. Partial HRSV G gene amplification and sequencing were performed as described by Botosso et al. [48].

### Phylodynamics of HRSV

Demographic dynamics based on HRSV gene genealogies (*i.e.*, phylodynamics) were estimated from partial G gene sequences (n = 1983) using the Bayesian Markov Chain Monte Carlo (MCMC) method implemented in the BEAST, v1.5.4 package (http://beast.bio.ed.ac.uk/ [91]. For phylodynamics studies the temporal relationship among all sequences was established by using sequences with known date of sampling of both HRSVA and HRSVB in respect to the HRSVA Long strain sampled in 1956 [48]. Bayesian MCMC analyses were performed using a relaxed molecular clock model (uncorrelated lognormal-distributed model - UCLD), which allows for variation in the substitution rate between monophyletic lineages. Analyses were independently performed using the general time-reversible (GTR) nucleotide substitution model, with a gamma-distributed among-site rate variation with four rate categories. Bayesian MCMC analyses were repeated using the constant size and exponential growth models in order to investigate the degree to which dating estimates are affected by the demographic model chosen. Each Bayesian MCMC analysis was run for 50 million states and sampled every 10,000 states. Posterior probabilities were calculated with a burnin of 2 million states and checked for convergence using the Tracer v1.4 program (http://beast.bio.ed.ac.uk/Tracer) with uncertainties depicted as 95% highest probability density (HPD) intervals. Bayesian skyline plots (BSL), which depict estimates for the relative viral genetic diversity (*Ne.g*) that relates to the relative change in the effective number of infections through time, were estimated for all HRSV genotypes. To measure the changes in growth rate (*r*) over time estimates were obtained with BEAST, v1.5.4 [91] from the best-fit demographic model for each genotype and analyzed over the sampling period. Maximum clade credibility (MCC) trees for HRSV genotypes were obtained by pooling five independent MCMC runs, each of which sampled from 20 million chains after a pre-burning period of 30 million chains, to ensure sampling from a stationary MCMC (inspected with Tracer). Analyses of selective pressures were performed by parsimonious reconstructions of the positively selected sites along the phylogenetic trees of the G protein of HRSVA and HRSVB using the ‘accelerated transformation’ (ACCTRAN) and the ‘delayed transformation’ (DELTRAN) methods implemented in MacClade v4.07 (MacClade for Mac OS X Rel. 4.07. 2005. Sunderland, MA: Sinauer Associates, Inc.), as previously described by Botosso et al. [48].

### Phylogeography of HRSV

Geographical origin of each sample was coded as a set of terminal unordered character states for each HRSV sequence, represented as a single capital letter. Analysis was performed using a standard continuous-time Markov chain (CTMC) model with the Bayesian stochastic search variable selection (BSSVS) procedure. The most parsimonious description of geographic spread was obtained by MCMC sampling from the plausible set of trees using BEAST v1.5.4 [91]. Entry dates of time stamped sequences were estimated with 95% Highest Probability Density (HPD) in the Tracer v1.4 program. Bayes factor (BF) analysis implemented in BEAST v1.5.4 was used to identify rates between two locations. During the discrete characters migration analyses, BF values above 3 were assumed to provide substantial strength, and BF above 30 to provide very strong evidence for migration events taking place. Nevertheless, because sequences were time-stamped, providing trees with dated nodes, information from the adjacency patterns in the MCC trees was sought to obtain evidence for genotype exchange from nodes with posterior probabilities near or above 90% (p≅0.9), when rates of exchange among localities had BF below 3. The FigTree v1.3.1 program (http://tree.bio.ed.ac.uk/software/figtree/) was used to visualize spatial and temporal information, and the virtual globe software Google Earth (http://earth.google.com).

### Cross-correlation analysis

To estimate the degree to which HRSV types and genotypes are correlated over time, we did a time series analysis. Data from the Bayesian skyline consisting of *Ne.g* over time generated with BEAST were synchronized by interpolation using polynomial interpolation in Origin v6.1052 software (1991–2000, Northampton, MA: OriginLab Corporation) and transformed into comparable time series. To establish whether a relationship exists between pairs of synchronous series, we computed correlation coefficients with in SPSS v. 11.0.4 (Chatfield 1975; SPSS for Mac Rel. 11.0.4. 2005. Chicago: SPSS Inc.) over a range of time lags of n = 365, n = 730, n = 999 days, that cover one up to three years, corresponding to time spans between seasonal-spaced cycle to minimize possible autocorrelation noise from data interpolation.

## Supporting Information

Figure S1 **Sampling of HRSV between the years 1956 and 2005.**
(EPS)

Figure S2 **Cross-correlation analysis of HRSV.** Cross-correlation coefficient as a function of the lag number set as the yearly cycle that HRSVA and HRSVB were displaced in time. A) Between 1989 to October 1999. B) Between November 1999 to 2006. A lag of 365 was used to discern cross correlation of one-year cycles between HRSV groups. Dotted lines (...) represent the likelihood bounds corresponding to a 95% confidence interval.
(EPS)

Figure S3 **Maximum clade credibility (MCC) phylogenies for HRSV genotypes.** MCC phylogenies are based on continents with branches colored according to the most probable location state of their descendent nodes.
(TIF)

Figure S4 **Google Earth ‘movies’ depicting inference results on the movement of HRSV genotypes around the globe.** These files can be directly uploaded to the Google Earth software (http://www.google.com/earth/index.html). For a global view of the spread of all HRSV genotypes in time load all movies at once. a)GA2 b)GA5 c)GB3 d) GB3 insert e)SAA1 f)SAB1 g)SAB3 h)GA6 i)GA7 j)GB4 k)SAB2.
(ZIP)

Table S1 **GenBank accession numbers of sequences used in this study.**
(DOC)

Table S2 **Details of samples used in the study.**
(DOCX)
