# Supplementary figures and images for: Phylodynamics and Dispersal of HRSV Entails Its Permanence in the General Population in between Yearly Outbreaks in Children
Source: PLoS One. 2012 Oct 15;7(10):e41953. doi: 10.1371/journal.pone.0041953 (PMC3471929; doi:10.1371/journal.pone.0041953)

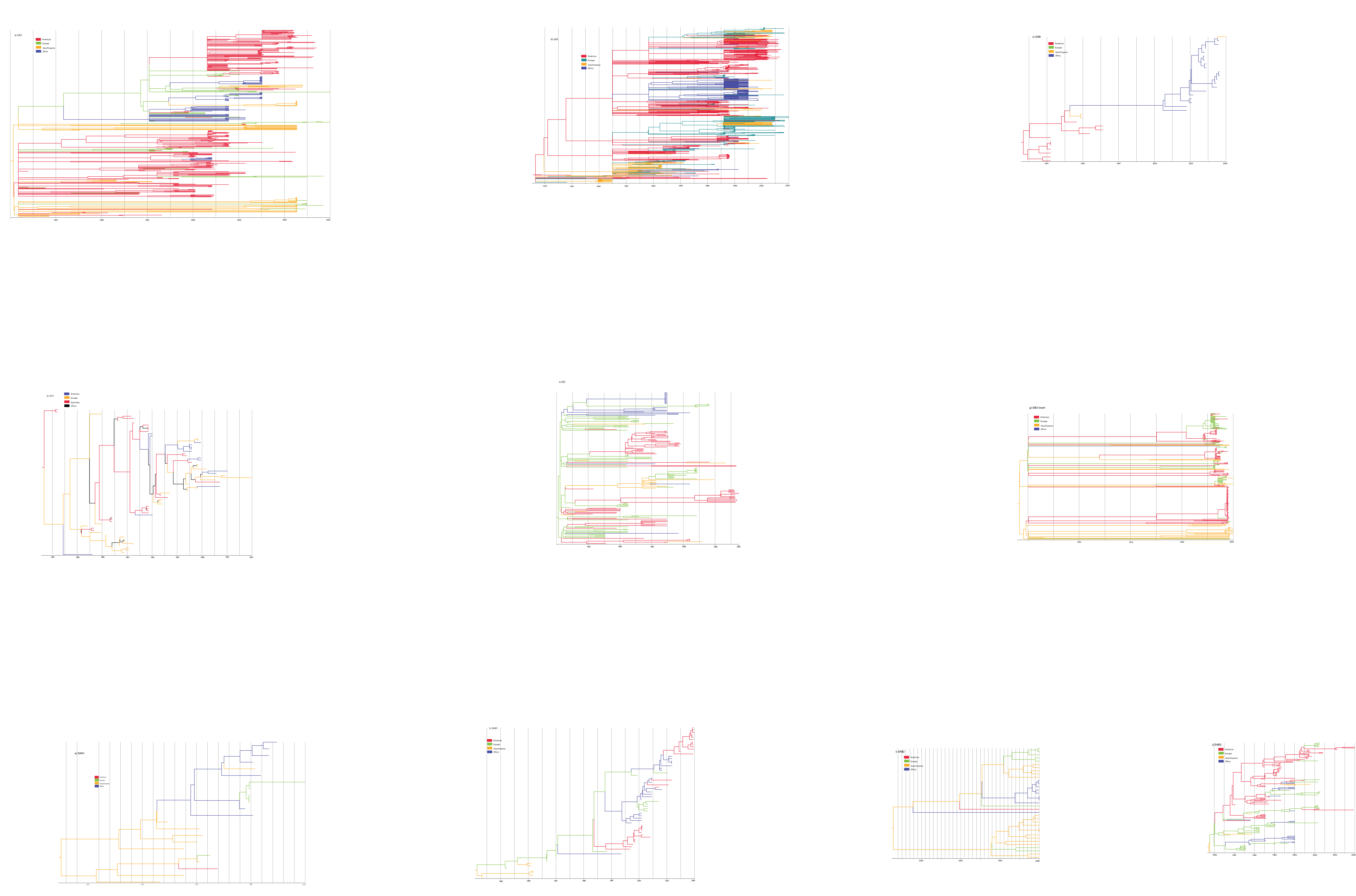

Supplement: Figure S3 — Maximum clade credibility (MCC) phylogenies for HRSV genotypes. MCC phylogenies are based on continents with branches colored according to the most probable location state of their descendent nodes. (TIF) [file pone.0041953.s003.tif]
