# Supplementary material for: Phylodynamics and Dispersal of HRSV Entails Its Permanence in the General Population in between Yearly Outbreaks in Children
Source: PLoS One. 2012 Oct 15;7(10):e41953. doi: 10.1371/journal.pone.0041953 (PMC3471929; doi:10.1371/journal.pone.0041953)
Supplement: Table S2 — Details of samples used in the study. (DOCX) [file pone.0041953.s006.docx]

**Table S2**. Details of samples used in the study.

| **Continent**  **Country** | **HRSV samples** | **Years** | **HRSVA** | **Genotypes**  **HRSVA** | **HRSVB** | **Genotypes**  **HRSVB** | **Reference** | | **Accession Number** |
| --- | --- | --- | --- | --- | --- | --- | --- | --- | --- |
| **Europe** |  |  |  |  |  |  |  | |  |
| Belgium | 112 | 1984-2002 | 112 | Total - 112  GA1,GA2,GA5,GA4 | - | - | Zlateva et al. 2004 [[1](#_ENREF_1)] | | AY343549 to AY343659 |
|  | 979 | 1996-2006 | 63 | Total – 63  GA1,GA2,GA5,GA4 | 142 | GB2, GB3, GB6, GB8, GB10, GB13 | Zlateva et al. 2007 [[2](#_ENREF_2)] | | DQ985156 to DQ985080 |
|  | 196 | 1982-2004 |  | - |  | GB1 to GB13 | Zlateva et al. 2005 [[3](#_ENREF_3)] | | AY751282 to AY751084 |
| Italy -  (Turin and Roma) |  | 1997 to 2006 | 59 | GA2, GA5 and only one BEA1 | 41 | - | Montieri et al. 2007 [[4](#_ENREF_4)] | | EU025187 to EU025245. |
| Spain | 39 | 1988 to 1993 | 39 | * | - | - | Garcia et al. 1994 [[5](#_ENREF_5)] | | Z33410 to Z33412  Z33414 to Z33419; Z33454 |
| Turkey | 5 | 2005/2006 | 5 | # | - | - | Midilli 2008 | DQ985748 to DQ985744 | |
| United Kingdon | 6 | 1989 | A | **SHL1, SHL-2, SHL-3, SHL-4, SHL-5 | - | - | Cane et al. [[6](#_ENREF_6)] | X73350.1 to  X73355.1 | |
|  | 29 | 1995-1996 | 15 | **SHL-1,3,4, SHL-2 | 14 | NP3 | Taylor et al. 2001 [[7](#_ENREF_7)] | AJ290196 to AJ290224 | |
| **Americas** |  |  |  |  |  |  |  | |  |
| Canada (Quebec)  Canada  (Winnipeg) | 249 | 2001/2002;  2002/2003 | 106 | GA2 (98), GA5 (7), 1 (GA7) | 94 | GB3 (76), SAB3 (14), SAB1 (4) | Gilca et al. 2006 [[8](#_ENREF_8)] | AY927387 to  AY927382  AY927413 to AY927401 | |
|  | 53 | 1994/1995 | 44 | GA2, GA3, GA5, GA7 | 9 | GB3, GB4 | Peret et al. 2000 [[9](#_ENREF_9)] | AF233904 to AF233908  AF233927 to AF263926 | |
|  |  |  |  |  |  |  |  |  | |

| **Continent**  **Country** | **HRSV samples** | **Years** | **HRSVA** | **Genotypes**  **HRSVA** | **HRSVB** | **Genotypes**  **HRSVB** | **Reference** | | **Accession Number** |
| --- | --- | --- | --- | --- | --- | --- | --- | --- | --- |
| **Americas** |  |  |  |  |  |  |  | |  |
| EUA  New York  Alabama, New York, Texas, Missouri  West Virginia | 204 | 1990/1991 to 1994/1995 | 123 | GA1 – GA5 | 81 | GB1-GB4 | Peret et al. 1998 [[10](#_ENREF_10)] | AF065258 to AF065250 | |
|  | 167 | 1994/1995 | 139 | GA1-GA3, GA5-GA7 | 28 | GB3-GB4 | Peret et al. 2000 [[9](#_ENREF_9)] | AF233900 to AF233903;  AF233910 to  AF233925;  AF233928 to AF233933 | |
|  | 6 | 1979, 1981, 1982, 1984, 1987, 1988 | 6 | * | - | - | Sullender et al. 1998 [[11](#_ENREF_11)] | AF065410 to AF065405 | |
| Brazil (Salvador) | 52 | 1999 | 13 | GA5, GA2, GA7, | 4 | GB3, SAB3 | Moura et al. 2004 [[12](#_ENREF_12)] | | AY472086 to AY472102 |
| Argentina | 7 | 1993 to 2001 | 7 | GA1, GA2, GA5 | - | - | Frabasile et al. 2003 [[13](#_ENREF_13)] | | AF516127 to  AF516125  AF516123 to  AF516121  AF516116 |
|  | 129 | 1995 to 2001 | 96 | Total - 37  GA2(54%),GA3,GA5 (32%), GA7 | 33 | Total – 20  GB3 (20%), GB4, SAB3 | Galiano et al. 2005 [[14](#_ENREF_14)] | | AY667096 to AY667068  AY672701 to AY672685 |
|  | 353 | 1999 to 2004 | 232 | GA2 (25) and GA5 (30) | 121 | - | Viegas et al. 2005 [[15](#_ENREF_15)] | | AY910822 to AY910769 |
|  | 353 | 1999 to 2004 | - | - | 47 | BA-like | Trento et al. 2006 [[16](#_ENREF_16)] | | DQ227408 to DQ227363 |
|  |  | 1999 | - | - | 3 | BA-like | Trento et al. 2003 [[17](#_ENREF_17)] | | AY333364 to AY333362 |
| **Continent**  **Country** | **HRSV samples** | **Years** | **HRSVA** | **Genotypes**  **HRSVA** | **HRSVB** | **Genotypes**  **HRSVB** | **Reference** | | **Accession Number** |
| **Americas** |  |  |  |  |  |  |  | |  |

| Uruguay | | | 37 | | 1987-1992 | 37 | GA1-GA3, GA5 | - | - | Garcia et al. 1994 [[5](#_ENREF_5)] | | Z33421 to Z33432; Z33494 |
| --- | --- | --- | --- | --- | --- | --- | --- | --- | --- | --- | --- | --- |
|  |  |  | 25 | | 1993-2001 | 25 | GA1-GA3, GA5 | - | - | Frabasile et al. 2003 [[13](#_ENREF_13)] | | AF516137 to AF516128  AF516125, AF516124  AF516120 to AF516117  AF516114 to  AF516107  AF448498 |
|  |  |  | 13 | | 1999; 2001 | - | - | 13 | SAB3, SAB2, URU1, URU2 | Blanc et al. 2005[[18](#_ENREF_18)] | | AY488806 to AY488794 |
| **Asia/Oceania** | | |  | |  |  |  |  |  |  | |  |
| Japan (Niigata) | | | 185 | | 11/2001 to 07/2004 | 92 | GA2, GA5, GA7 | 63 | SAB3, GB3, Insert | Sato et al. 2005 [[19](#_ENREF_19)] | | AB175823 to AB175814 |
| Japan (Sapporo and Tokyo, Osaka) | | | 109 | | 1980 to 2002 | 49 | GA1, GA4, GA5, GA7, JA1 | 47 | GB1, GB3, GB4, SAB3, Jab1 | Kuroiwa et al. 2005 [[20](#_ENREF_20)] | | AB161422 to AB161377 |
| China -  (Beijing,  Lanzhou, Changchu) | | | 83 | | 1990/91,  2000/01, 2003/04 | 76 | GA2 (74/76), GA3 (1/76), GA5 (1/76) | 4 | GB3 | Zhang et al. 2007 [[21](#_ENREF_21)] | | DQ289649.1 to  DQ289597.1 |
| China  (Zhejiang) | | | 4 | | 2004 | 4 | # | - | - | - | | AY728170 to AY728167 |
| India | | | 48 | | 10/2001 to 12/2004 | 37 | GA5 (29), GA2 (8) | 11 | BA (11) | Parveen et al. 2006 [[22](#_ENREF_22)] | DQ248894 to DQ248930 | |
|  |  |  | - | | 2005 to 2007 | - | - | - | # | Bharaj 2008 | EU368623 to EU368650 | |
| **Continent**  **Country** | | | **HRSV samples** | | **Years** | **HRSVA** | **Genotypes**  **HRSVA** | **HRSVB** | **Genotypes**  **HRSVB** | **Reference** | | **Accession Number** |
| **Asia/Oceania** | | |  | |  |  |  |  |  |  | |  |
| Korea | 257 | | | 11/1990-02/1999 | | 188 | * | 65 | * | Choi and Lee, 2000 [[23](#_ENREF_23)] | | AF193338 to AF193304 |
| New Zealand | | 144 | | | 1967 and  1981-2004 | 106 | UA, GA2, GA3, SAA1,GA7, GA4,GA6,NZA1,  GA5 | 38 | GB1,GB2,  GB3,GB4, GB5, GB12, GB13, NZB1,NZB2,UA | Matheson et al. 2006 [[24](#_ENREF_24)] | | DQ171878 to DQ171735 |
| **Africa** | |  | | |  |  |  |  |  |  | |  |
| Mozambique | | 275 | | | 1998/1999 | 10 | * | 20 | * |  | | AF309684 to AF309655 |
| Kenya | 397 | | | 1999 to 2001 | | 319 | 98  GA2, GA5 | 57 | 11  SAB1, GB3 Insert | Scott et al. 2004 [[25](#_ENREF_25)] | | AY660684 to AY660667,  AY524663 to  AY660573 |
|  | 25 | | | 2002-2003 | | 21 | GA2, GA5 | 4 | SAB1, GB3 Insert | Scott et al. 2006 [[26](#_ENREF_26)] | | AY773286 to AY773288  AY773290 to  AY773301 |
| South Africa | 91 | | | 2000-2001 | | 42 | Total - 27  GA2,SAA1,GA5 | 7 | SAB1,SAB3,GB3 | Madhi et al. 2003 [[27](#_ENREF_27)] | | AY146444 to  AY146411 |
|  | 8 | | | 2000-2001 | | 8 | # | 19 | # | Agenbach,E., Madhi,S.A. and Venter,M. Venter et al. 2001[28] | | AY226537 to AY226511 |
|  | 225 | | | 1997-2000 | | 144 | Total - 65  GA5,GA7,GA2,  SAA1 | 81 | Total – 38  GB3, GB4,SAB1, SAB2,SAB3 |  | | AF348802 to AF348826 |

*These genotypes were not genotyped by the authors

**This classification is based in SH gene data. SHl-1, 3, 4 corresponds to GA3, GA2 and GA7 genotypes, SHL-2 corresponds to GA5 genotype, and SLH-5 corresponds to GA1 genotype

# Direct submission to Gen Bank. Unpublished data

$ No sequences were obtained in 1998-2000 and 2003-2004 seasons

GB3 Insert – GB3 with 60-nucleotide insertion

**REFERENCES**

1. Zlateva KT, Lemey P, Vandamme AM, Van Ranst M (2004) Molecular evolution and circulation patterns of human respiratory syncytial virus subgroup a: positively selected sites in the attachment g glycoprotein. Journal of virology 78: 4675-4683.

2. Zlateva KT, Vijgen L, Dekeersmaeker N, Naranjo C, Van Ranst M (2007) Subgroup prevalence and genotype circulation patterns of human respiratory syncytial virus in Belgium during ten successive epidemic seasons. Journal of clinical microbiology 45: 3022-3030.

3. Zlateva KT, Lemey P, Moes E, Vandamme AM, Van Ranst M (2005) Genetic variability and molecular evolution of the human respiratory syncytial virus subgroup B attachment G protein. Journal of virology 79: 9157-9167.

4. Montieri S, Puzelli S, Ciccozzi M, Calzoletti L, Di Martino A, et al. (2007) Amino acid changes in the attachment G glycoprotein of human respiratory syncytial viruses (subgroup A) isolated in Italy over several epidemics (1997-2006). Journal of medical virology 79: 1935-1942.

5. Garcia O, Martin M, Dopazo J, Arbiza J, Frabasile S, et al. (1994) Evolutionary pattern of human respiratory syncytial virus (subgroup A): cocirculating lineages and correlation of genetic and antigenic changes in the G glycoprotein. Journal of virology 68: 5448-5459.

6. Cane PA, Matthews DA, Pringle CR (1991) Identification of variable domains of the attachment (G) protein of subgroup A respiratory syncytial viruses. The Journal of general virology 72 ( Pt 9): 2091-2096.

7. Taylor GS, Vipond IB, Caul EO (2001) Molecular epidemiology of outbreak of respiratory syncytial virus within bone marrow transplantation unit. Journal of clinical microbiology 39: 801-803.

8. Gilca R, De Serres G, Tremblay M, Vachon ML, Leblanc E, et al. (2006) Distribution and clinical impact of human respiratory syncytial virus genotypes in hospitalized children over 2 winter seasons. The Journal of infectious diseases 193: 54-58.

9. Peret TC, Hall CB, Hammond GW, Piedra PA, Storch GA, et al. (2000) Circulation patterns of group A and B human respiratory syncytial virus genotypes in 5 communities in North America. The Journal of infectious diseases 181: 1891-1896.

10. Peret TC, Hall CB, Schnabel KC, Golub JA, Anderson LJ (1998) Circulation patterns of genetically distinct group A and B strains of human respiratory syncytial virus in a community. The Journal of general virology 79 ( Pt 9): 2221-2229.

11. Sullender WM, Mufson MA, Prince GA, Anderson LJ, Wertz GW (1998) Antigenic and genetic diversity among the attachment proteins of group A respiratory syncytial viruses that have caused repeat infections in children. The Journal of infectious diseases 178: 925-932.

12. Moura FE, Blanc A, Frabasile S, Delfraro A, de Sierra MJ, et al. (2004) Genetic diversity of respiratory syncytial virus isolated during an epidemic period from children of northeastern Brazil. Journal of medical virology 74: 156-160.

13. Frabasile S, Delfraro A, Facal L, Videla C, Galiano M, et al. (2003) Antigenic and genetic variability of human respiratory syncytial viruses (group A) isolated in Uruguay and Argentina: 1993-2001. Journal of medical virology 71: 305-312.

14. Galiano MC, Luchsinger V, Videla CM, De Souza L, Puch SS, et al. (2005) Intragroup antigenic diversity of human respiratory syncytial virus (group A) isolated in Argentina and Chile. Journal of medical virology 77: 311-316.

15. Viegas M, Mistchenko AS (2005) Molecular epidemiology of human respiratory syncytial virus subgroup A over a six-year period (1999-2004) in Argentina. Journal of medical virology 77: 302-310.

16. Trento A, Viegas M, Galiano M, Videla C, Carballal G, et al. (2006) Natural history of human respiratory syncytial virus inferred from phylogenetic analysis of the attachment (G) glycoprotein with a 60-nucleotide duplication. Journal of virology 80: 975-984.

17. Trento A, Galiano M, Videla C, Carballal G, Garcia-Barreno B, et al. (2003) Major changes in the G protein of human respiratory syncytial virus isolates introduced by a duplication of 60 nucleotides. The Journal of general virology 84: 3115-3120.

18. Blanc A, Delfraro A, Frabasile S, Arbiza J (2005) Genotypes of respiratory syncytial virus group B identified in Uruguay. Archives of virology 150: 603-609.

19. Sato M, Saito R, Sakai T, Sano Y, Nishikawa M, et al. (2005) Molecular epidemiology of respiratory syncytial virus infections among children with acute respiratory symptoms in a community over three seasons. Journal of clinical microbiology 43: 36-40.

20. Kuroiwa Y, Nagai K, Okita L, Tsutsumi H (2004) Genetic variability and molecular epidemiology of respiratory syncytial virus subgroup a strains in Japan determined by heteroduplex mobility assay. Journal of clinical microbiology 42: 2048-2053.

21. Zhang Y, Xu W, Shen K, Xie Z, Sun L, et al. (2007) Genetic variability of group A and B human respiratory syncytial viruses isolated from 3 provinces in China. Archives of virology 152: 1425-1434.

22. Parveen S, Broor S, Kapoor SK, Fowler K, Sullender WM (2006) Genetic diversity among respiratory syncytial viruses that have caused repeated infections in children from rural India. Journal of medical virology 78: 659-665.

23. Choi EH, Lee HJ (2000) Genetic diversity and molecular epidemiology of the G protein of subgroups A and B of respiratory syncytial viruses isolated over 9 consecutive epidemics in Korea. The Journal of infectious diseases 181: 1547-1556.

24. Matheson JW, Rich FJ, Cohet C, Grimwood K, Huang QS, et al. (2006) Distinct patterns of evolution between respiratory syncytial virus subgroups A and B from New Zealand isolates collected over thirty-seven years. Journal of medical virology 78: 1354-1364.

25. Scott PD, Ochola R, Ngama M, Okiro EA, Nokes DJ, et al. (2004) Molecular epidemiology of respiratory syncytial virus in Kilifi district, Kenya. Journal of medical virology 74: 344-354.

26. Scott PD, Ochola R, Ngama M, Okiro EA, James Nokes D, et al. (2006) Molecular analysis of respiratory syncytial virus reinfections in infants from coastal Kenya. The Journal of infectious diseases 193: 59-67.

27. Madhi SA, Venter M, Alexandra R, Lewis H, Kara Y, et al. (2003) Respiratory syncytial virus associated illness in high-risk children and national characterisation of the circulating virus genotype in South Africa. Journal of clinical virology : the official publication of the Pan American Society for Clinical Virology 27: 180-189.

28. Venter M, Madhi SA, Tiemessen CT, Schoub BD (2001) Genetic diversity and molecular epidemiology of respiratory syncytial virus over four consecutive seasons in South Africa: identification of new subgroup A and B genotypes. The Journal of general virology 82: 2117-2124.
